# Genotype-Specific Vector Competence of *Aedes albopictus* for Japanese Encephalitis Virus Genotypes I, III, and V

**DOI:** 10.3390/v17101323

**Published:** 2025-09-29

**Authors:** Bo-Ram Yun, Ji-Young Kwon, Dongmi Kwak, Hee Il Lee

**Affiliations:** 1 Division of Vectors and Parasitic Diseases, Korea Disease Control and Prevention Agency, Cheongju 28159, Republic of Korea; boram2183@korea.kr (B.-R.Y.); kjiy31@korea.kr (J.-Y.K.); 2 College of Veterinary Medicine, Kyungpook National University, Daegu 41569, Republic of Korea; 3 Institute for Veterinary Biomedical Science, College of Veterinary Medicine, Kyungpook National University, Daegu 41566, Republic of Korea

**Keywords:** vector competence, *Aedes albopictus*, Japanese encephalitis virus, genotypes

## Abstract

Japanese encephalitis virus (JEV), a mosquito-borne flavivirus, poses a significant public health threat in Asia. Although *Culex* species are primary vectors, the role of *Aedes albopictus* in JEV transmission has gained attention under changing ecological conditions. This study evaluated the vector competence of *Ae. albopictus* for three JEV genotypes: I (GI), III (GIII), and V (GV). Laboratory-reared *Ae. albopictus* were orally challenged with each genotype, and infection rate (IR), dissemination rate (DR), head–thorax positivity rate (HTR, proxy for potential transmission), and transmission rate (defined as saliva positivity) were assessed at 7 and 14 days post-infection (dpi). *Ae. albopictus* showed marked genotype-specific differences. By 14 dpi, GV had the highest DR (100.0%) and HTR (71.7%), with viral RNA detected in 36.7% of TR. GIII showed moderate competence (76.9% DR, 39.3% HTR), but low TR (6.6%). In contrast, GI-infected mosquitoes exhibited minimal infection and negligible transmission, with viral RNA rarely detected beyond the midgut. These findings indicate that *Ae. albopictus* is highly competent for transmitting JEV genotype V and moderately for genotype III, but not genotype I, under laboratory conditions. This highlights its potential role in the transmission dynamics of emerging JEV genotypes and underscores the need for continued surveillance.

## 1. Introduction

Japanese encephalitis virus (JEV), a mosquito-borne flavivirus, is the leading cause of viral encephalitis in Asia, responsible for an estimated 68,000 cases and over 10,000 deaths annually, predominantly among children in endemic regions [1]. The virus is maintained in a zoonotic transmission cycle involving Culex mosquitoes, particularly *Culex tritaeniorhynchus*, and vertebrate amplifying hosts, such as pigs and ardeid birds [2]. Human infections are incidental but can lead to outbreaks under favorable ecological and climatic conditions [3,4,5,6].

Among the five recognized JEV genotypes (GI–GV), genotype III (GIII) was the first to be identified, initially isolated in Japan in 1935 [7,8]. Genotype II (GII) and genotype V (GV) were subsequently reported in the 1950s from India and Malaysia, respectively [9,10], while genotype IV (GIV) was detected in Indonesia in 1979. Genotype I (GI), now the dominant strain across much of Asia, emerged in East Asia in the late 1970s [2,11,12,13].

Historically, GIII was the predominant genotype circulating in East and Southeast Asia. However, over the past two decades, GI has largely displaced GIII as the dominant strain in many regions [14]. Notably, GV, once considered extinct following its initial isolation in Malaysia, re-emerged in 2009 when it was detected in *Cx. tritaeniorhynchus* in China [15]. In the Republic of Korea (ROK), GV was first identified in 2010 in *Cx. bitaeniorhynchus*, with subsequent detections in other *Culex* species, including *Cx. orientalis* in 2020, indicating its sustained circulation [14,15,16]. GIV has also been reported, mainly in Indonesia and parts of Oceania, but its limited geographic distribution and relatively low public health impact have resulted in sparse investigation [17].

Although *Culex* species remain the principal vectors of JEV, accumulating evidence suggests that *Aedes albopictus* may also contribute to its transmission, particularly in ecologically altered or urban environments [18,19,20]. Commonly known as the Asian tiger mosquito, *Ae. albopictus* is a highly adaptable species with a global distribution facilitated by international trade and climate change. In ROK, *Ae. albopictus* is already widely distributed nationwide [21]. JEV RNA has been detected in field-collected *Ae. albopictus* in ROK [22]. While repeated isolations of JEV from *Ae. albopictus* have been reported in Asia, the primary vectors remain *Culex* species, and the epidemiological significance of *Ae. albopictus* in natural transmission cycles is still unclear [23,24,25]. The established competence of *Ae. albopictus* for transmitting other arboviruses—including dengue, chikungunya, and Zika—warrants further investigation into its potential role in JEV transmission [19,20,23,26,27].

Vector competence, the intrinsic ability of a mosquito to acquire, sustain, and transmit a pathogen, varies with both mosquito species and viral genotype. Prior studies have demonstrated that susceptibility to JEV can vary among mosquito species, populations, and viral genotypes, highlighting the importance of investigating local vector competence for both primary and potential supplementary vectors [18,20,21,28]. However, comparative data on *Ae. albopictus* responses to different JEV genotypes remain limited. In particular, the ability of *Ae. albopictus* to support replication and transmission of the re-emerging GV genotype is poorly characterized. This gap is especially critical given the recent predominance of GV in ROK, underscoring the need for localized assessments of vector competence.

Given the ongoing genotype shifts in JEV and the expanding ecological range of *Ae. albopictus*, it is essential to evaluate its potential role in transmitting diverse JEV genotypes. In this study, we assessed the vector competence of laboratory-reared *Ae. albopictus* for JEV GI, GIII, and GV. Using oral infection assays, we evaluated genotype-specific differences in infection, dissemination, and transmission at multiple time points post-infection. These findings aim to enhance understanding of genotype-dependent transmission dynamics and inform future risk assessments and vector control strategies in the context of evolving JEV ecology.

## 2. Materials and Methods

### 2.1. Mosquitoes

*Ae. albopictus* (F43 generation), originally collected from Incheon, ROK, were used for experimental infections. Following field collection, the colony was continuously maintained under laboratory conditions. Mosquitoes were reared at 27 ± 1 °C, 70 ± 5% relative humidity, and a 12:12 h light–dark photoperiod. Adults were provided with 10% sucrose ad libitum, and females were blood-fed weekly using an artificial membrane feeding system (Hemotek Ltd., Accrington, UK). Each genotype was tested in three independent biological replicates, using newly prepared infectious blood meals and separate cohorts of mosquitoes.

### 2.2. Production and Titration of JEV Strains for Vector Competence Assays

JEV GI was isolated from *Cx. tritaeniorhynchus* in 2005 [29], GIII from the same species in 1994 [29], and GV from *Cx. orientalis* in 2020 [16] (Table 1). Viral stocks were propagated in Vero cells as previously described [19,30]. Briefly, each strain was cultured in Eagle’s Minimum Essential Medium (MEM; Welgene, Gyeongsan, Republic of Korea) supplemented with 10% fetal bovine serum (FBS; Thermo Fisher Scientific, Waltham, MA, USA), 100 U/mL penicillin, and 100 μg/mL streptomycin at 37 °C in a humidified incubator (Vision Scientific Co.,Ltd., Daejeon, Republic of Korea) with 5% CO_2_ for 3 days in T75 flasks. Viral replication was confirmed by the presence of cytopathic effects (CPE) observed under a light microscope (ZEISS, Oberkochen, Germany). For virus titration, BHK-21 cells were seeded in six-well plates(SPL, Pocheon, Republic of Korea) 1 day prior to infection. Serial 10-fold dilutions of each viral stock were prepared in MEM containing 2% FBS and 1% penicillin–streptomycin (P/S), and 300 μL of each dilution was inoculated into individual wells. Following 1 h of incubation at 37 °C with 5% CO_2_, the inoculum was replaced with 4 mL of overlay medium containing 0.5% agarose, 2% FBS, and 1% P/S in MEM. After 5 days of incubation at 37 °C, the cells were fixed with 4% paraformaldehyde for 30 min and stained with 1% crystal violet. Plaques were counted, and viral titers are expressed as plaque-forming units per milliliter (PFU/mL).

### 2.3. Oral Infection of JEV

All infection assays were conducted in a Biosafety Level 3 (BSL-3) facility (Permit No. KCDC-18-3-04) at the Division of Vectors and Parasitic Diseases, Korea Disease Control and Prevention Agency (KDCA). Female *Ae. albopictus* mosquitoes (5–7 days old) were deprived of sucrose for 24 h prior to exposure. Infectious blood meals were prepared by mixing defibrinated sheep blood (Kisan Bio Co., Ltd., Seoul, Republic of Korea) with virus suspension at a 2:1 ratio, supplemented with ATP to a final concentration of 10 nM to stimulate feeding. Viral titers in the blood meals were adjusted to 6.5 × 10^6^–1.0 × 10^7^ PFU/mL for GI, 1.2 × 10^6^–1.0 × 10^7^ PFU/mL for GIII, and 1.0 × 10^6^–1.0 × 10^7^ PFU/mL for GV. Mosquitoes were allowed to feed overnight in a dark incubator at 27 ± 1 °C using a Hemotek membrane feeding system (Hemotek Ltd., Blackburn, UK). Fully engorged females were selected and maintained under standard rearing conditions (27 ± 1 °C, 70 ± 5% relative humidity, 12:12 h light–dark cycle) for the remainder of the experiment.

### 2.4. Mosquito Dissection and Saliva Collection

To evaluate infection dynamics, mosquitoes were dissected at 7 and 14 days post-infection (dpi) in the BSL-3 insectary under controlled conditions (27 ± 1 °C, 70 ± 5% relative humidity). For each individual, the midgut, legs and wings, and head–thorax were carefully separated and transferred into individual microcentrifuge tubes (SPL, Pocheon, Republic of Korea) containing 300 μL of MEM supplemented with 2% FBS and a ceramic homogenization bead. These tissues were used to assess infection (midgut), dissemination (legs and wings), and head–thorax positivity as a proxy for potential salivary gland invasion. At 14 dpi, saliva was also collected to assess transmissibility. Cold-anesthetized mosquitoes had their legs and wings removed, and their proboscis was inserted into a 10 μL droplet of phosphate-buffered saline (PBS) held within a cut pipette tip under the same environmental conditions (27 ± 1 °C, 70 ± 5% RH). After 30 min of salivation, saliva droplets were collected. All dissected tissues and saliva samples were stored at −80 °C until analysis.

Infection rate (IR), dissemination rate (DR), and transmission rate (TR) were determined based on the detection of viral RNA in specific tissues. IR was defined as the proportion of mosquitoes with viral RNA detected in the midgut, indicating successful initial infection. DR was calculated as the proportion of infected mosquitoes with viral RNA in the legs and wings, representing viral dissemination beyond the midgut barrier. Head–thorax positivity rate (HTR) was defined as the proportion of mosquitoes with viral RNA detected in the head–thorax, serving as a proxy for potential transmission.

In accordance with the accepted definition, TR is defined strictly as the proportion of mosquitoes with viral RNA detected in saliva, reflecting actual transmission potential. In this study, HTR is presented alongside TR as a complementary measure, since saliva-based assays may underestimate transmission due to methodological limitations.

### 2.5. RNA Extraction and qRT-PCR Detection of JEV

All tissue samples, excluding saliva, were homogenized using the Precellys^®^ Evolution homogenizer (Bertin Technologies, Montigny-le-Bretonneux, France) with two cycles of bead beating at 7500 rpm for 30 s each. Homogenates were centrifuged at 13,000 rpm for 1 min, and 30 μL of the supernatant was transferred to a new tube for RNA extraction. Total RNA was extracted using the Clear-S™ Total RNA Extraction Kit (Invirustech, Gwangju, Republic of Korea) according to the manufacturer’s instructions.

JEV RNA targeting the non-structural protein 5 (NS5) gene was detected using the Clear-MD^®^ Flavivirus Real-Time RT-PCR Detection Kit (Invirustech, Gwangju, Republic of Korea). The qRT-PCR conditions were as follows: reverse transcription at 45 °C for 10 min; enzyme inactivation at 95 °C for 10 min; followed by 40 cycles of amplification consisting of denaturation at 95 °C for 10 s, annealing at 60 °C for 15 s, extension at 72 °C for 10 s, and signal acquisition at 80 °C for 15 s. A melting curve analysis was subsequently performed to confirm product specificity: denaturation at 95 °C for 30 s, annealing at 70 °C for 30 s, followed by gradual heating to 95 °C in 0.5 °C increments every 30 s. Samples with a cycle threshold (Ct) value ≤ 40 and a melting peak between 82 °C and 88 °C were considered positive for JEV RNA. Quantification was calibrated using a standard curve generated from a Vircell JEV RNA quantified control (MBC134-R; AMPLIRUN Japanese Encephalitis RNA control, Granada, Spain, 2024) using 10-fold serial dilutions (10^5^–10^3^ copies per reaction); the resulting standard-curve data are provided in Supplementary Figure S1.

### 2.6. Viral Titration by TCID_50_ Assay

To quantify infectious virus, samples from the legs–wings, body, and head–thorax were collected at 7 and 14 dpi. Each tissue homogenate (30 μL) was used for titration via the 50% tissue culture infectious dose (TCID_50_/mL) assay using BHK-21 cells. BHK-21 cells were seeded into 96-well plates at a density of 1.0 × 10^4^ cells/well in 200 μL of MEM supplemented with 5% FBS and 1% penicillin–streptomycin (P/S), and incubated overnight at 37 °C. Serial 10-fold dilutions of each homogenate were prepared in MEM containing 2% FBS, and 100 μL of each dilution was added to five replicate wells. Plates were incubated at 37 °C for 5 days, after which CPE were visually assessed. Cells were subsequently fixed and stained with crystal violet to confirm CPE. Viral titers were calculated using the Reed–Muench method based on the number of CPE-positive wells at each dilution [3].

### 2.7. Statistical Analysis

All statistical analyses were performed using GraphPad Prism version 5.0 (GraphPad Software, San Diego, CA, USA). Comparisons of viral RNA loads between time points within each tissue were assessed using the nonparametric Mann–Whitney U test. For comparisons across multiple groups (e.g., among JEV genotypes), the Kruskal–Wallis test, followed by Dunn’s multiple comparisons test, was applied. Differences in HTR (proxy for potential transmission) and TR (defined as saliva positivity) between 7 and 14 dpi were analyzed for each genotype using Fisher’s exact test. PTR (population transmission rate) was calculated as the proportion of saliva-positive mosquitoes among blood-fed survivors. A *p*-value < 0.05 was considered statistically significant.

## 3. Results

### 3.1. Genotype-Dependent Differences in the Vector Competence of Ae. albopictus

The vector competence of *Ae. albopictus* for three JEV genotypes (GI, GIII, GV) was assessed based on IR, DR, HTR, and TR (saliva positivity) at 7 and 14 dpi (Table 2). The data revealed pronounced genotype-specific differences. GV-infected mosquitoes displayed the highest competence at 14 dpi, with a DR of 100.0% and HTR of 71.7%. However, the TR, defined as saliva positivity, was 36.7%, indicating that although many mosquitoes showed evidence of potential salivary gland invasion, only about one-third were capable of viral excretion. Mosquitoes infected with GIII showed intermediate competence. At 14 dpi, DR reached 76.9% and HTR was 39.3%, but the corresponding TR was only 6.6%, suggesting inefficient release of virus into saliva despite moderate dissemination and potential salivary gland infection.

In contrast, GI-infected mosquitoes exhibited minimal competence. At 14 dpi, dissemination was not observed beyond the midgut in most individuals, and the TR was only 4.0%, confirming negligible transmission potential for this genotype in *Ae. albopictus*. Together, these results demonstrate that while HTR provides an indicator of potential transmission, TR based on saliva positivity represents the conservative and accepted measure of actual transmission capacity, which varied markedly among JEV genotypes.

### 3.2. Temporal Dynamics of Viral Dissemination and Transmission

**Figure 1:**
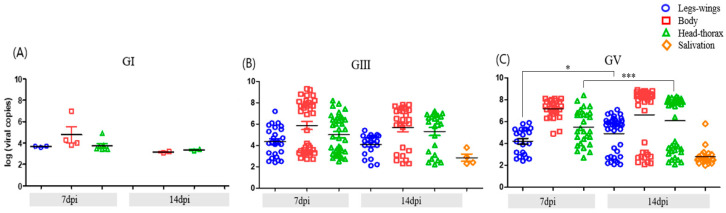
Viral RNA loads in *Ae. albopictus* tissues (midgut, legs–wings, head–thorax) and saliva at 7 and 14 dpi with JEV GI (**A**), GIII (**B**), and GV (**C**). Head–thorax RNA positivity was used to calculate transmission rate (TR), while saliva RNA positivity was evaluated separately as an indicator of actual viral excretion. Each dot represents an individual mosquito, and horizontal bars indicate the mean ± standard error of the mean (SEM). Statistical differences between 7 and 14 dpi within each tissue were assessed using the Mann–Whitney U test (* *p* < 0.05, *** *p* < 0.001).

Temporal analysis revealed efficient progression of GV infection within mosquito tissues between 7 and 14 dpi. A marked increase in viral RNA detection in both the head–thorax region and saliva was observed at 14 dpi, indicating successful viral escape from tissue barriers and a high probability of transmission. In GIII-infected mosquitoes, dissemination to secondary tissues was already apparent by 7 dpi and remained relatively stable thereafter, with only a modest increase over time. In contrast, GI infection showed no substantial progression during the same period, remaining largely restricted to the midgut. These patterns underscore distinct temporal dynamics in viral replication and dissemination across JEV genotypes (Figure 1).

### 3.3. Infectious Viral Titers in Ae. albopictus

To determine the presence of infectious virus, TCID_50_ assays were performed on mosquito body, legs–wings, and head–thorax samples at 7 and 14 dpi (Table 3). Infectious JEV was consistently detected in GIII-infected mosquitoes. Mean viral titers in the body were 2.39 ± 0.10 log_10_ TCID_50_/mL (n = 9) at 7 dpi and 2.30 ± 0.08 log_10_ TCID_50_/mL (n = 5) at 14 dpi. Although viral dissemination to the head–thorax was observed in a limited number of mosquitoes, the corresponding titers remained low, 2.33 and 2.35 log_10_ TCID_50_/mL at 7 and 14 dpi, respectively.

### 3.4. Transmission Potential of Ae. albopictus at the Population Level

**Figure 2:**
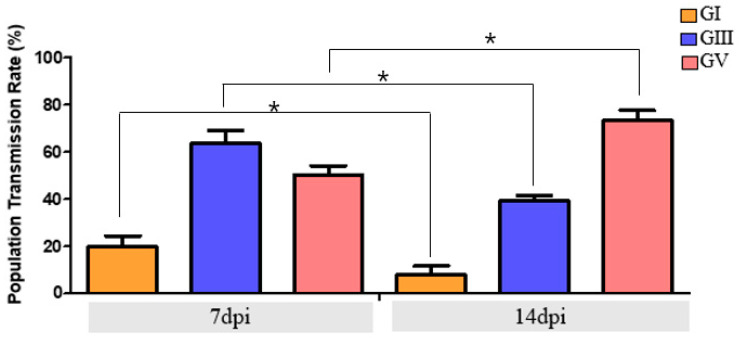
Comparison of population transmission rates (PTR) of *Ae. albopictus* orally infected with JEV GI, GIII, and GV at 7 and 14 dpi. PTR was calculated as the proportion of mosquitoes with detectable JEV RNA in the head–thorax (HTR, proxy for potential transmission) or saliva (TR, actual transmission). While HTR reflects possible salivary gland invasion, TR represents the accepted definition of transmission rate based on viral detection in saliva. Statistical differences in PTR-TR between 7 and 14 dpi were evaluated for each genotype using Fisher’s exact test (* *p* < 0.05).

To evaluate the population-level transmission potential of *Ae. albopictus*, viral RNA was quantified in both the head–thorax region (head–thorax positivity rate, indicating potential transmission) and saliva (reflecting actual transmission) (Figure 2). At 14 dpi, GV-infected mosquitoes exhibited the highest HTR (71.7%) based on head–thorax positivity; however, the TR was 36.7%, providing the most accurate indicator of transmission. GIII-infected mosquitoes showed an HTR of 39.3%, but only 6.6% TR, reflecting a strong barrier to viral excretion. GI-infected mosquitoes exhibited minimal competence, with just 4.0% TR, confirming negligible transmission potential. These comparisons highlight that while HTP provides a proxy measure of potential salivary gland invasion, TR based on saliva positivity represents the conservative and accepted indicator of true transmission capacity. These findings indicate a markedly reduced ability of GI to escape midgut and salivary barriers, further highlighting the limited transmission potential of this genotype in *Ae. albopictus*.

## 4. Discussion

This study provides novel insights into the genotype-specific vector competence of *Ae. albopictus* for JEV, with particular emphasis on the recently re-emerged GV. Under controlled laboratory conditions, *Ae. albopictus* supported the infection, dissemination, and transmission of JEV GV at levels comparable to, or exceeding, those of GIII, a historically dominant strain. In contrast, GI, currently the predominant circulating strain in East Asia, exhibited markedly reduced vector competence, with limited midgut infection and negligible transmission potential [30,31].

In line with the accepted definition, TR was defined strictly as the proportion of mosquitoes with detectable JEV RNA in saliva [32,33]. To provide additional context on viral dissemination within the vector, we also evaluated population transmission rates based on HTR as a proxy for potential transmission. HTR reflects possible salivary gland invasion but may overestimate actual transmission capacity, since the presence of viral RNA in the head–thorax does not guarantee viral excretion. Conversely, TR offers the most conservative and reliable indicator of true transmission, although it may underestimate potential because forced salivation methods can fail to detect low-level expectoration [31,34,35,36,37,38,39]. Accordingly, HTR and TR should be interpreted as complementary rather than contradictory indicators of vector competence [36].

Our results demonstrated that GV-infected mosquitoes had both high HTR (71.7%) and substantial TR (36.7%), indicating efficient dissemination and partial but effective escape into saliva. For GIII, HTR was 39.3%, but TR was only 6.6%, suggesting a pronounced bottleneck at the level of viral release into saliva. For GI, the TR was just 4.0%, confirming negligible transmission potential. These genotype-dependent differences highlight that viral replication and dissemination kinetics within the mosquito strongly influence ultimate transmission potential [18,19].

A notable finding was the poor vector competence of *Ae. albopictus* for GI. Despite its predominance in current human and animal infections, GI rarely disseminated beyond the midgut in infected mosquitoes, and transmission was undetectable. This suggests that GI exhibited low replication efficiency in *Ae. albopictus* and instead relied primarily on other vectors, such as *Cx. tritaeniorhynchus,* for efficient transmission. Alternatively, genotype-specific immune responses, such as enhanced activation of RNA interference or other antiviral pathways in *Ae. albopictus*, may suppress GI replication. These findings highlight the complexity of genotype–vector compatibility, which is shaped by the interplay between viral genetics and vector physiology. This interplay is also strongly influenced by ecological and climatic factors [27]. Consistent with this observation, the number of GI-positive mosquitoes was markedly lower at 14 dpi (n = 7), despite a total of 106 individuals being engorged with GI-infected blood meals across three independent replicates. In contrast, 38 and 29 mosquitoes remained positive for GIII and GV, respectively, at 7 dpi. This uneven sample size reflects the inherently reduced replication and persistence of GI in *Ae. albopictus* rather than insufficient experimental design, further supporting genotype-specific differences in viral replication dynamics.

Temporal infection dynamics further emphasized the differences among the genotypes. GV demonstrated rapid progression from infection to head–thorax positivity (TR) and saliva positivity between 7 and 14 dpi, consistent with high replication efficiency and systemic dissemination. GIII exhibited early dissemination by 7 dpi but showed limited progression thereafter, while GI remained largely restricted to the midgut throughout. These trends were corroborated by our temporal analysis (Section 3.2), which showed a marked increase in viral RNA in the head–thorax and saliva of GV-infected mosquitoes at 14 dpi, indicating effective escape from tissue barriers. In contrast, GIII showed minimal change after initial dissemination, and GI failed to progress meaningfully over time. Together, these findings demonstrate that intra-vector replication kinetics are a critical determinant of genotype-specific transmission potential [2,19]. Such genotype dynamics are also shaped by repeated introductions across regions, as shown by molecular studies indicating frequent incursions of JEV from Southeast and East Asia into Japan [37]. Consistent with saliva assay results, only 4.0% of GI-infected mosquitoes were saliva-positive, further confirming the negligible transmission potential of this genotype in *Ae. albopictus*.

Taken together, our findings indicate that *Ae. albopictus* exhibits only low to moderate transmission competence for JEV when evaluated using saliva positivity as the benchmark. This conservative interpretation aligns with entomological surveillance in the ROK where JEV detections in *Ae. albopictus* have been rare (two positive pools out of >40,000 tested) [16,22]. Although experimental infections have demonstrated that *Ae. albopictus* is a competent laboratory vector for more than 20 arboviruses [37,38], field evidence supports its major role only for chikungunya virus and, to a lesser extent, dengue and Zika viruses. For JEV, while repeated isolations from *Ae. albopictus* and other mosquitoes have been reported in Asia [24,25,26,27,28,29,30,31,32,33,34,35,36,37,38,39], these should be interpreted with caution. Virus isolation alone is insufficient to incriminate a species as a natural vector; this requires supporting evidence of host feeding patterns, natural infection dynamics, and efficient transmission to vertebrate hosts [38]. Thus, while *Culex* species remain the principal vectors of JEV, the supplementary role of *Ae. albopictus*—particularly for GV—in urban and peri-urban environments cannot be dismissed [18,37,39,40].

This study has several limitations. First, all experiments were conducted under controlled laboratory conditions using a laboratory-reared mosquito colony; therefore, environmental temperature fluctuations, host availability, microbiota composition, and vector age—factors known to influence vector competence—were not captured. Future investigations should validate these findings under semi-field or field conditions and elucidate the molecular mechanisms that underlie genotype-specific susceptibility in *Ae. albopictus*.

Second, although inoculated viral titers differed slightly among JEV genotypes, all infectious blood meals were maintained within the biologically relevant range of 10^6^–10^7^ PFU/mL commonly used in vector-competence studies; thus, minor variation within this window is unlikely to have substantially influenced infection outcomes [34]. Third, although saliva positivity (TR) is the most direct measure of transmission, it may underestimate potential due to technical limitations of forced salivation, whereas head–thorax (HTR) may overestimate it; thus, both indicators should be interpreted as complementary rather than contradictory [31,33,34]. Finally, to further assess external transmissibility, future studies will include mammalian infection/challenge experiments to validate transmission potential in vertebrate hosts.

In conclusion, our results demonstrate that *Ae. albopictus* is a competent but limited vector for JEV GV and GIII, while showing negligible competence for GI, under laboratory conditions. Importantly, this represents the first evidence of GV transmission by *Ae. albopictus*, underscoring that this globally distributed and ecologically adaptable mosquito could act as a supplementary vector under certain ecological contexts. These findings highlight the need for continued monitoring of JEV genotype–vector interactions, while carefully distinguishing laboratory competence from field incrimination, to better assess their potential impact on future transmission dynamics in the context of ecological change and climate-driven expansion [18,39].

## Figures and Tables

**Table 1:** Details of Japanese Encephalitis Virus Strains Used for Experimental Infections.

Virus Strain (Genotype)	Year	Source	Artificial Virus Titer (PUF/mL)	GenBank Accession No.	Reference
K05-GS (GI)	2005	*Cx. tritaeniorhynchus*	6.5 × 10^6^–1.0 × 10^7^	FJ938223	Yun SM et al., 2010 [29]
K94A071 (GIII)	1994	*Cx. tritaeniorhynchus*	1.2 × 10^6^–1.0 × 10^7^	FJ938217	Yun SM et al., 2010 [29]
Sangju (GV)	2020	*Cx. orientalis*	1.0 × 10^6^–1.0 × 10^7^	MZ868506	Seo MG et al., 2021 [16]

**Table 2:** Vector Competence of *Ae. albopictus* for JEV GI, GIII, and GV at 7 and 14 Dpi. IR, DR, HTR and TR of *Ae. albopictus* orally infected with JEV GI, GIII, and GV at 7 and 14 dpi. Each rate was determined by the detection of JEV RNA in specific mosquito body parts: body (IR), legs and wings (DR), and head–thorax (HTR). TR (Saliva positivity) at 14 dpi is presented separately as a direct indicator of viral excretion. JEV detection was performed using qRT-PCR.

Genotype of JEV	Total Number Processed	7 dpi			14 dpi			
		IR	DR	HTR	IR	DR	HTR	TR
GI	106	7/56 (12.5%) ^a^	3/7 (42.9%) ^a^	11/56 (19.6%) ^a^	0/50 (N/A) ^a^	0/0 (N/A) *	2/50 (4.0%) ^a^	2/50 (4.0%) ^a^
GIII	119	38/58 (65.5%) ^b^	26/38 (68.4%) ^a^	37/58 (63.8%) ^b^	26/61 (42.6%) ^b^	20/26 (76.9%) ^a^	24/61 (39.3%) ^b^	4/61 (6.6%) ^a^
GV	116	29/56 (51.8%) ^b^	22/29 (75.9%) ^a^	28/56 (50.0%) ^b^	39/60 (65.0%) ^b^	39/39 (100.0%) ^a^	43/60 (71.7%) ^b^	22/60 (36.7%) ^b^

Percentages represent IR, DR, HTR, and TR at 7 and 14 dpi. Superscript letters (^a^, ^b^) indicate statistically significant differences among JEV genotypes for each parameter and time point, based on two-tailed Fisher’s exact tests with Bonferroni correction (*p* < 0.05). * For DR, values are calculated only from IR-positive mosquitoes. “N/A” indicates that dissemination rate could not be determined due to the absence of infected individuals (i.e., zero denominator).

**Table 3:** Infectious virus titers (log_10_ TCID_50_/mL) of JEV in *Ae. albopictus* tissues at 7 and 14 dpi.

JEV Genotype	Dpi	Body Titer (log_10_ TCID_50_/mL) ± SD (n)	Leg-Wings Titer (log_10_ TCID_50_/mL) ± SD (n)	Head-Thorax Titer (log_10_ TCID_50_/mL) ± SD (n)
GI	7	N/A	N/A	N/A
	14	N/A	N/A	N/A
GIII	7	2.39 ± 0.10 (9)	N/A	2.33 (1)
	14	2.30 ± 0.08 (5)	N/A	2.35 (1)
GV	7	2.39 ± 0.11 (15)	N/A	2.35 (1)
	14	2.67 ± 0.23 (19)	2.43 (1)	2.52 ± 0.15 (21)

Mean ± standard deviation (SD) of virus titers detected in the body, legs-wings, and head–thorax of *Ae. albopictus* infected with JEV genotypes GI, GIII, and GV. Virus titration was performed using the TCID_50_ assay on BHK-21 cells. “N/A” indicates data not available due to absence of positive samples or insufficient material.

## Data Availability

All data are in the manuscript.

## Supplementary Materials

The following supporting information can be downloaded at: https://www.mdpi.com/article/10.3390/v17101323/s1, Figure S1: tandard curve for the JEV NS5 RT-qPCR generated from a quantified JEV RNA control.

## Abbreviations

*Ae.*	*Aedes* (mosquito genus)
BHK	Baby hamster kidney-21 cells
Ct	Cycle threshold (qRT-PCR)
CPE	Cytopathic effect
DR	Dissemination rate
dpi	Days post-infection
GI	Genotype I Japanese encephalitis virus
GII	Genotype II Japanese encephalitis virus
GIII	Genotype III Japanese encephalitis virus
GIV	Genotype IV Japanese encephalitis virus
GV	Genotype V Japanese encephalitis virus
IR	Infection rate
JEV	Japanese encephalitis virus
KDCA	Korea Disease Control and Prevention Agency
KCDC	Korea Centers for Disease Control and Prevention
NS5	NON-STRUCTURAL PROTEIN 5 of flaviviruses
P/S	Penicillin–streptomycin
PTR	Population transmission rate
ROK	Republic of Korea
TCID_50_	50% Tissue-culture infectious dose
TR	Transmission rate
