# Supplementary material for: Genotype-Specific Vector Competence of Aedes albopictus for Japanese Encephalitis Virus Genotypes I, III, and V
Source: Viruses. 2025 Sep 29;17(10):1323. doi: 10.3390/v17101323 (PMC12567606; doi:10.3390/v17101323)

Supplementary Data:

Figure S1. Standard curve for the JEV NS5 RT-qPCR generated from a quantified JEV RNA control (Vircell, Granada, Spain, Cat no. MBC134-R). Ten-fold serial dilutions spanning 10^5–10^3 copies per reaction were tested. Linear regression of Ct versus $\log_{10}$(copies) yielded $y = -3.5867x + 46.542$ with $R^2 = 0.997$, corresponding to an amplification efficiency of ~90% ($E = 10^{(-1/slope)} - 1$). The linear dynamic range for quantification was defined as 10^5–10^3 copies per reaction; values below this range were interpreted qualitatively according to predefined Ct/Tm criteria.

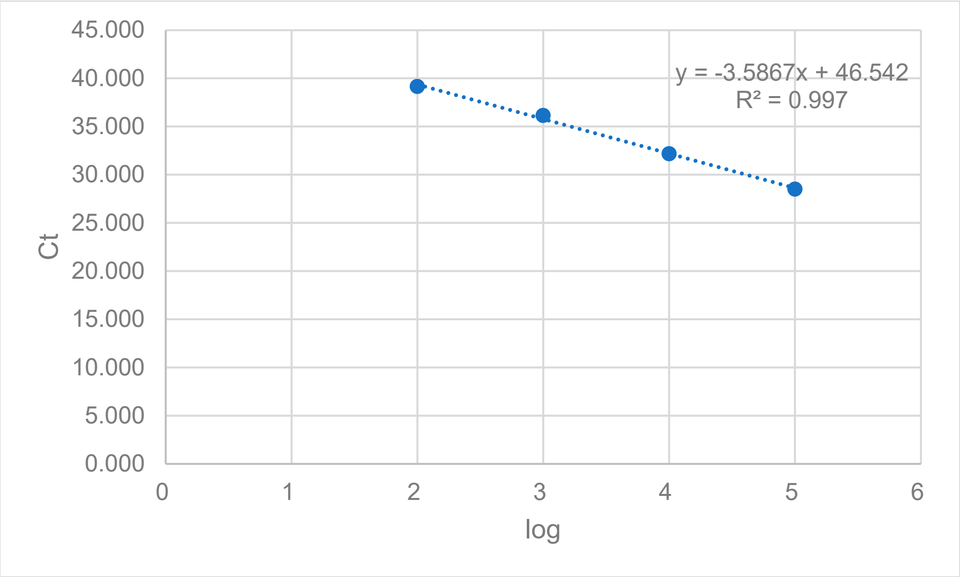

Supplement: Supplementary file 1 [file viruses-17-01323-s001.zip › viruses-3837602-supplementary.pdf]
